# A novel treatment of pediatric bilateral condylar fractures with lateral dislocation of the temporomandibular joint (TMJ) using transfacial pinning

**DOI:** 10.1080/23320885.2023.2242498

**Published:** 2023-08-03

**Authors:** Kerry A. Morrison, Roberto L. Flores

**Affiliations:** Hansjörg Wyss Department of Plastic Surgery, NY University Langone Health, New York, NY, USA

**Keywords:** Pediatric facial trauma, pediatric mandibular fractures, pediatric condylar fractures, pediatric symphyseal fractures, transfacial pinning, craniofacial surgery

## Abstract

A 3-year-old patient sustained a tripartite mandibular fracture, including bilateral condylar fractures with lateral dislocation of the left condyle and symphyseal fracture. Staged lower jaw reconstruction with closed reduction of the laterally dislocated condyle, transfacial pinning between the mandibular angles, MMF using circummandibular wiring and intermaxillary fixation screws was performed.

## Introduction

Pediatric facial trauma management remains a therapeutic challenge within plastic and reconstructive surgery. Notably, nearly 50 percent of pediatric facial fractures involve the mandible with the majority comprising condylar fractures, often with a concomitant symphyseal fracture [1–7]. Fractures of the condyle are more common in children than in adults because the highly vascularized pediatric condyle and thin neck are poorly resistant to impact forces [8,9]. In the adult patient population, the morbidity of condylar-symphyseal facial fractures is well known [10]. Indeed, combined condylar-symphyseal fractures in adults can lead to widening of the lower face, malocclusion, and limited oral excursion [11]. Although these challenges affect similarly affected children, the pediatric condyles are integral to mandibular growth, raising concern for surgical manipulation [12]. Furthermore, the presence of tooth buds may limit or preclude the ability to apply rigid fixation to the mandible [7,13]. However, the pediatric condyle’s inherent ability to remodel surpasses that of the adult, a characteristic which can be potentially leveraged during condylar fracture repair [14–16].

Nevertheless, there remains a paucity of literature on the treatment of these pediatric condylar-symphyseal fractures [7,17]. The central challenge is addressing the laterally displaced mandible due to the limited options to hold the displaced TMJ into reduction and still allowing for oral excursion to occur while maintaining rigid fixation to the remainder of the jaw fractures. In the absence of bony fixation, the action force of the masseter muscle will maintain the fracture fragments in their laterally displaced position. With regards to the appropriate surgical approach to the laterally dislocated temporomandibular joint (TMJ), Zide and Kent have specifically identified this injury as an indication for open reduction [18]. However, the risks to TMJ pathology in children affected by this fracture pattern, with or without open reduction is an area of concern. Combined condylar-symphyseal fractures can have serious implications for the TMJ ankylosis, facial growth, facial symmetry, long-term dental development, and occlusal status in children [19]. The incidence of TMJ ankylosis with or without growth retardation is reported in 1 to 7 percent of condylar fractures; however, this risk for ankylosis is even higher with bilateral condylar fractures, in children between the ages of 2 and 5 years, delayed treatment, or prolonged maxillomandibular fixation (MMF) course [8,9,20,21]. The tripartite mandible fracture (bilateral condyle and symphysis) has been described in both children and adults and can add another dimension of complexity due to the need of controlling multiple fracture segments [22–25].

Herein, this case report describes a novel technique for the management of pediatric bilateral condylar fractures with symphyseal fracture, and a laterally dislocated TMJ while preserving the developing teeth. Given the inherently limited options for rigid fixation in the growing pediatric mandible, a transfacial Steinman pin was used to provide semirigid fixation, and prevent lateral displacement of the TMJ.

## Case presentation

**Figure 1.**
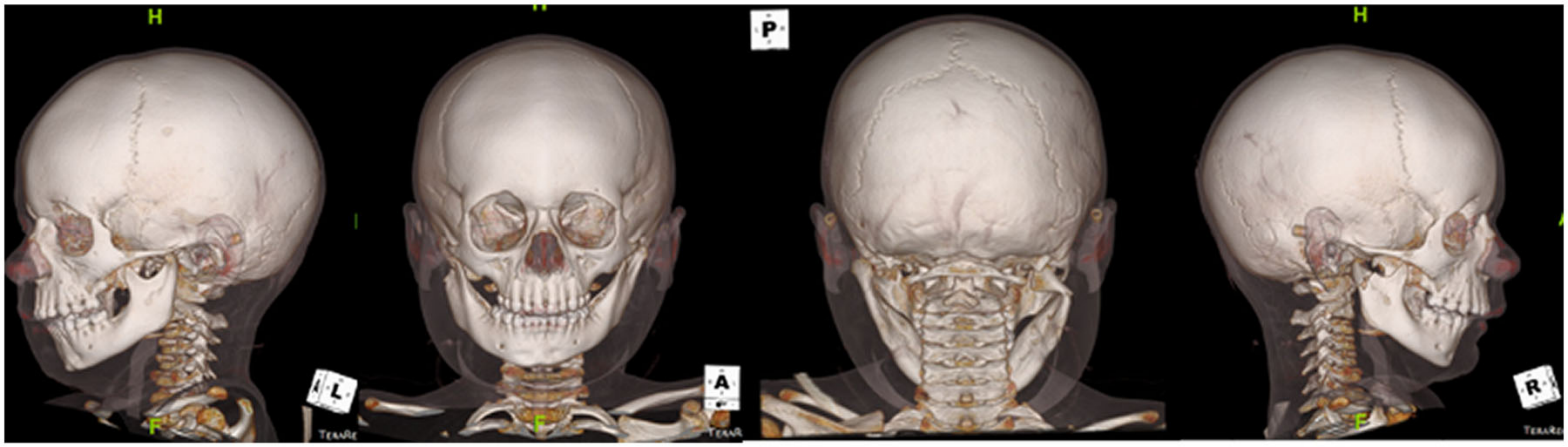
CT craniomaxillofacial findings revealed a complex, tripartite mandibular fracture, including bilateral condylar fractures with lateral dislocation of the left condyle and a symphyseal fracture. Specifically, there was a comminuted left condylar fracture with medial and inferior displacement of the fracture fragments as well as lateral and inferior dislocation of the intact lateral aspect of the condyle; near complete right subcondylar fracture with lateral apex angulation as well as medial and inferior dislocation of the right mandibular condyle; a mildly distracted oblique left parasymphyseal fracture; retrognathism; and, associated perimandibular soft tissue edema with mild subcutaneous edema on the right, and a 1.0 cm hematoma adjacent to the left mandibular condyle.

The patient was a healthy 3-year-old male, who was an unrestrained passenger in a golf cart accident. Physical examination was notable for panfacial edema with no soft tissue injuries, very limited oral excursion, and an intact facial nerve bilaterally. Computed tomographic (CT) craniomaxillofacial findings revealed a tripartite mandibular fracture, including bilateral condylar fractures with lateral dislocation of the left condyle and a symphyseal fracture (Figure 1). There was a complete right condylar neck fracture with lateral apex angulation as well as medial and inferior dislocation of the right mandibular condyle. The symphyseal fracture was associated with lateral displacement of the mandibular angle, bilaterally. Physical exam included bilateral lateral crossbite, retrognathia and an open bite deformity. The remainder of the patient’s facial architecture was intact, the patient’s cervical spine was cleared both clinically and radiographically, and there were no other physical injuries noted.

The operative plan consisted of a staged lower jaw reconstruction including closed reduction of the fracture with transfacial pinning and placement of circummandibular wiring as well as intermaxillary fixation screws for maxillomandibular fixation.

## Procedure

**Figure 2.**
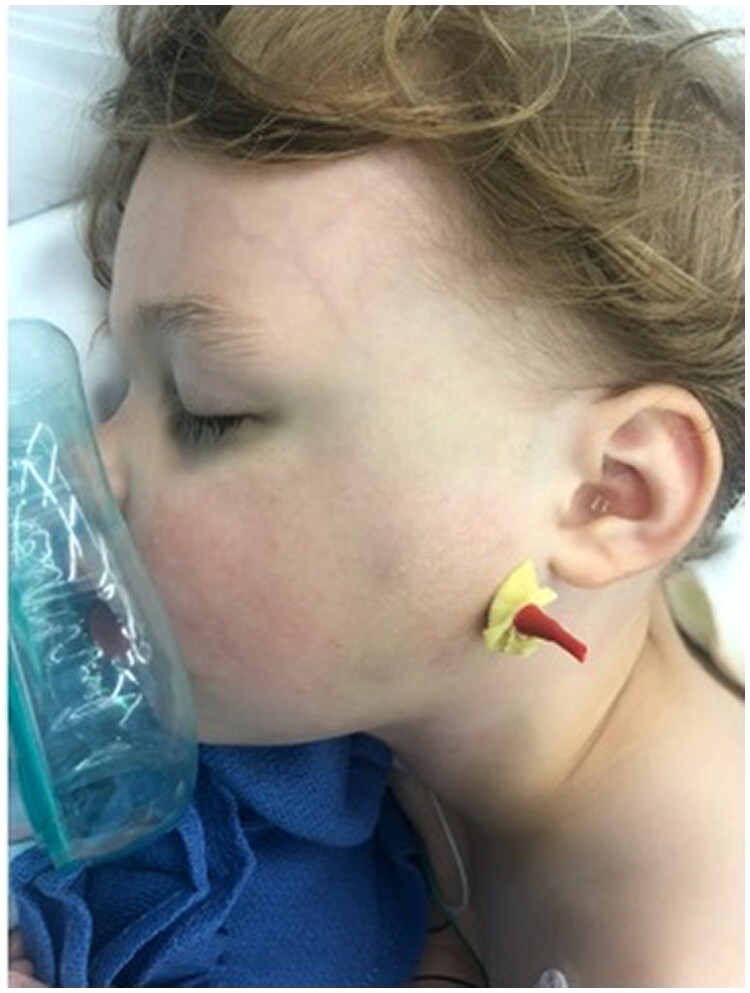
Clinical photograph intra-operatively showing the 2.8 mm threaded Steinman pin placed from left mandibular angle to right mandibular angle with a red rubber catheter with a xeroform dressing placed over the external portion of the pin for protection.

After right nasal endotracheal tube insertion with general anesthesia was achieved, attention was drawn to closed reduction of the fracture, as significant widening of the bigonial width and lateral dislocation of the left TMJ was a consequence of this tripartite fracture. Firm medial pressure at the mandibular angle was required to relocate the condyle on the left side and reduce the lower facial width. Appropriate reduction was confirmed by resolution of the anterior crossbite when the patient was brought into occlusion. Furthermore, temporomandibular joint was externally palpated with the jaw both open and closed, confirming reduction of the laterally dislocated left condyle. 1% lidocaine with 1:100,000 epinephrine was then injected into the skin overlying the left angle of the mandible. After 10 min elapsed, a 15-blade scalpel was used to make a puncture in the lower aspect of the cheek, and blunt dissection was used to reach the left angle of the mandible. A 2.8 mm threaded Steinman pin was engaged into left mandibular angle and carefully advanced to the right mandibular angle in a transfacial trajectory. Care was taken to avoid the tongue and endotracheal tube. This pin was advanced until the right angle of the mandible was penetrated however the tip of the pin remained within the soft tissue of the right cheek. This Steinman pin was cut, and a red rubber catheter with a xeroform dressing was placed over the external portion of the pin for protection (Figure 2).

Maxillomandibular fixation was then applied by placing intermaxillary fixation screws (KLS Martin Group, Tuttlingen, Germany) at the superior aspect of the pyriform aperture bilaterally. Dissection was performed in a subperiosteal plane, and pre-operative CT imaging was referenced to confirm location of the deciduous teeth and tooth buds prior to placement of the screws. Then, an 18-gauge spinal needle was used to thread a 26-gauge steel wire posterior and then anterior to the mandible on the right and left side, respectively. This looped 26-gauge steel wire was carefully placed through the intermaxillary fixation screws, and the maxillomandibular fixation was applied by twisting the wire. Appropriate occlusion was noted at the conclusion of the procedure.

## Post-operative course & follow-up

**Figure 3.**
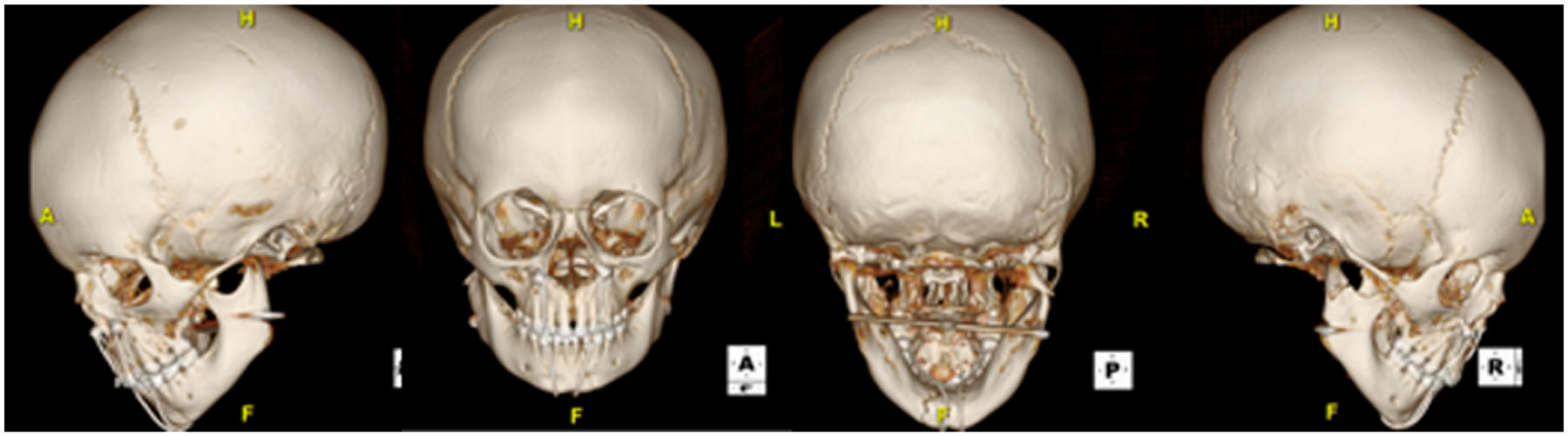
The immediate post-operative CT maxillofacial scan revealed anatomic reduction of the fracture Sites.

**Figure 4.**
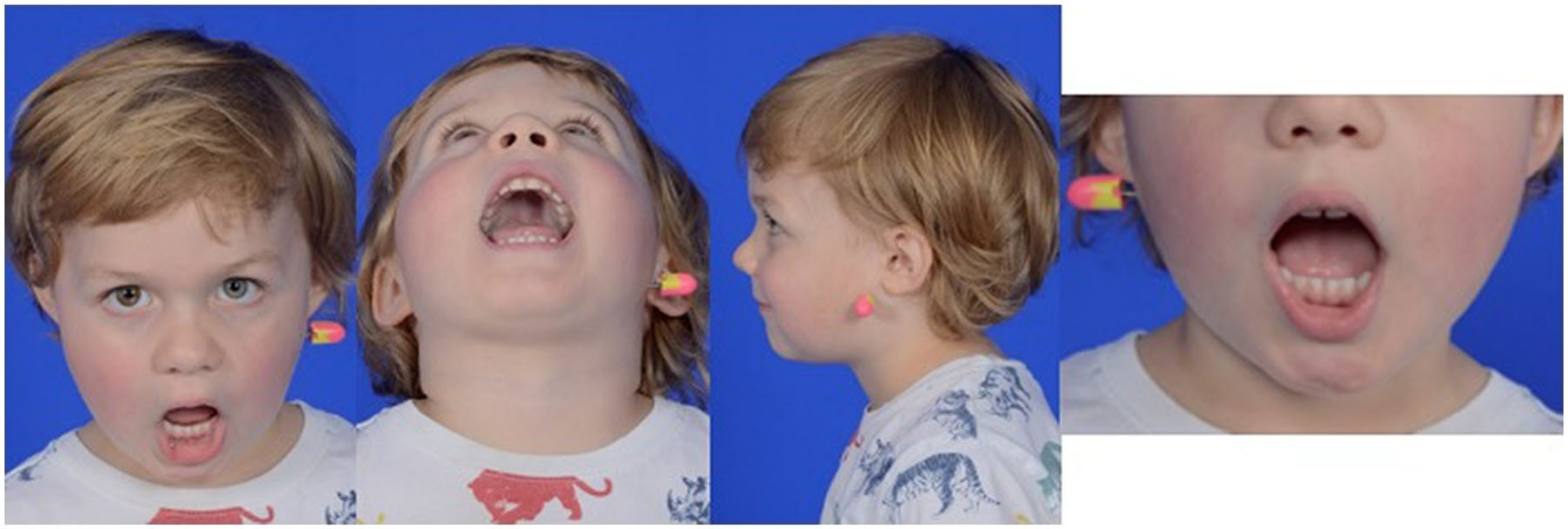
On physical examination at this 2-month follow up time, the patient was noted to have facial symmetry, class I occlusion, full oral excursion, and very minimal scarring at the left transfacial pin placement site.

**Figure 5.**
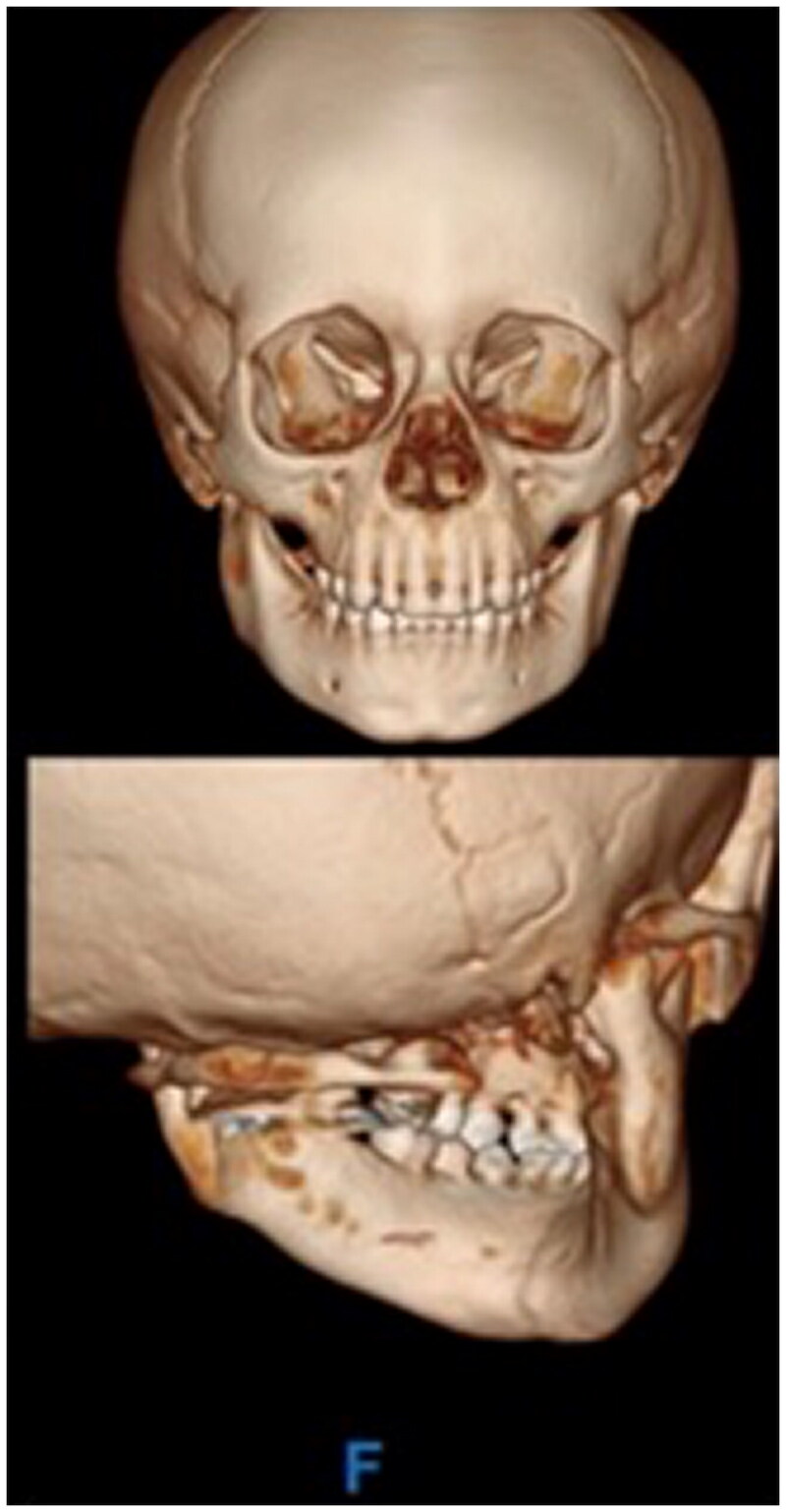
The follow-up CT maxillofacial scan at 5-months post-initial surgery demonstrated relocation, remodeling, and bony healing. Specifically, the left TMJ is restored and relocated, the right condylar fracture has remodeled and is in nearly anatomic pre-injury location, and the symphyseal fracture has healed.

**Figure 6.**
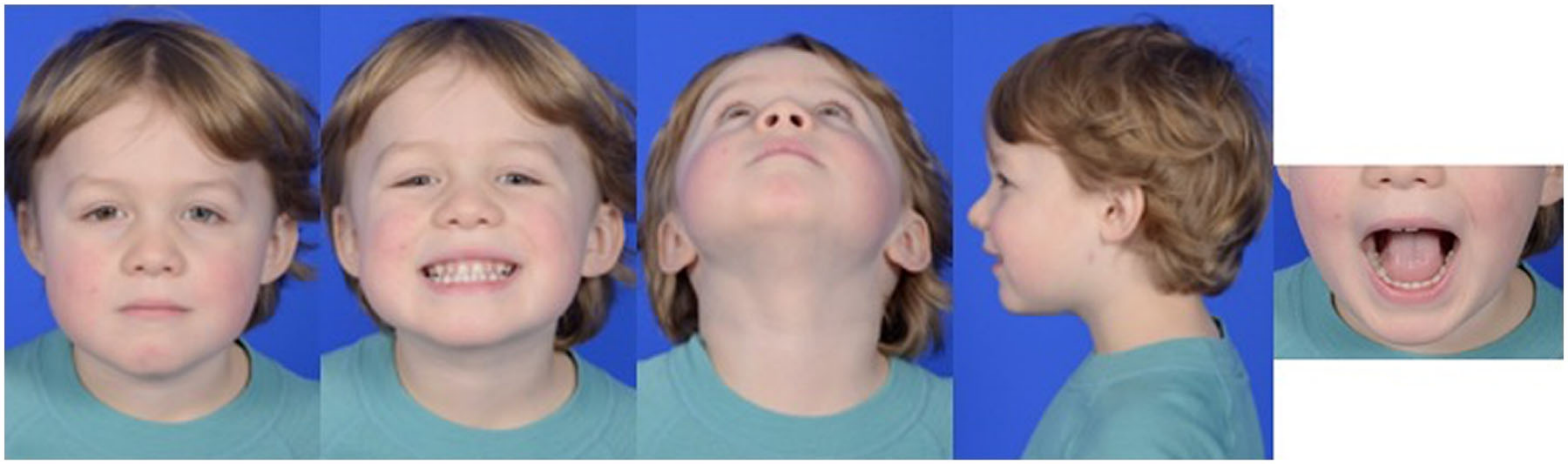
The patient’s clinical photographs at 5-month follow up showing facial symmetry, class I occlusion (pre-injury normal occlusion), full oral excursion, and nearly imperceptible scarring at the left transfacial pin placement site.

Post-operatively, the patient was placed on a liquid diet, and remained inpatient for monitoring overnight. The immediate post-operative CT craniomaxillofacial scan revealed anatomic reduction of the left TMJ and the mandibular angles with persistent medial dislocation of the right condyle (Figure 3). The transfacial Steinman pin site was protected at all times with either a red rubber catheter or small foam covering. The patient was discharged on post-operative day 1 from the hospital. He tolerated the maxillomandibular fixation very well with no complaints of pain, and had appropriate oral intake on a full liquid diet for the first two post-operative weeks with routine interval appointments in the clinic office while the maxillomandibular fixation was in place. The circummandibular wiring and intermaxillary fixation screws were surgically removed using a limited sulcus incision on post-operative day 14. During this procedure, occlusion was noted to be class I, without lateral crossbite and stable on jaw excursion. Range of motion physical therapy to the lower jaw was then initiated and the diet was advanced to soft foods. In the subsequent weeks, lower jaw excursion was noted to be increasing and without pain or shift from the midline. The transfacial Steinman pin was removed at 8 weeks after the initial repair. Oral excursion was measured at this time at the level of incisors as 27 mm. The patient restarted a pre-injury diet one week after transfacial Steinman pin removal. On physical examination at this 2-month follow up time, the patient was noted to have facial symmetry, class I occlusion, full oral excursion, and very minimal scarring at the left transfacial pin placement site (Figure 4). A follow-up computed tomography scan taken 5 months after the initial surgery demonstrated stable reduction of the left TMJ, remodeling of the right condylar fracture to near anatomic position, and radiographic healing of the symphyseal fracture (Figure 5). Physical exam demonstrated facial symmetry, class I occlusion with no retrognathia, full oral excursion with no deviation of the lower jaw, full facial nerve function and a small scar at the left transfacial pin placement site (Figure 6). The patient had returned to his normal diet, and had no pain on mastication. There is no retained hardware of the jaw and the developing teeth were surgically untouched by this surgical treatment plan.

## Discussion

Pediatric patients under the age of 5 years old account for only 5.6 percent of all facial fractures [26]. To date, consensus and clear evidence-based data on a comprehensive management protocol for pediatric mandibular fractures does not exist, especially in younger children, as the presence of tooth buds and deciduous teeth complicate interosseous fixation [24,27]. There are no treatment guidelines on the treatment of pediatric bilateral condylar fractures with concomitant symphyseal fracture. Furthermore, the surgical treatment of the laterally dislocated TMJ in younger children is also lacking. The current evidence is in favor of conservative management with closed reduction [9,28–31] however, open reduction internal fixation techniques of condylar fractures in children have also been reported [11,29,31–33].

This case report describes a transfacial pinning technique for the management of pediatric bilateral condylar fractures with symphyseal fracture and a laterally dislocated TMJ in a patient less than 5 years of age. Previously, the senior author reported the use of a transfacial Steinman pin along with maxillomandibular wiring for the treatment of pediatric bilateral condylar fractures with concomitant symphyseal fracture in a 4-year-old female patient with no complications [14]. In the case presented in this report, the transfacial pin was used to maintain reduction of the laterally dislocated left condylar fracture. It is notable, that the laterally dislocated TMJ is indication for open reduction as per criteria detailed by Zide and Kent [18]. This report demonstrates that the transfacial pin technique can be successfully applied to young children with a laterally dislocated TMJ resulting in stable reduction of the condyle, return to full range of jaw motion without pain, restoration of a normal diet and radiographic healing of all fractures. Furthermore, these two case reports provide supportive evidence for a novel treatment modality to treat tripartite bilateral condylar fractures in young children, a fracture pattern in which surgical indications are lacking.

In condylar-symphyseal fractures akin to this patient’s tripartite mandible, it is key to understand that without bony fixation, the unopposed action of the masseter muscle will maintain the fracture fragments in their laterally displaced location [14]. The utility of the transfacial Steinman pin is to provide semi-rigid fixation, and to prevent lateral displacement of the mandibular angles during bony healing. This technique mitigates any injury to adult tooth buds in the pediatric patient population, as the pin is placed through the angle of the mandible, obviating any need for screw or plate placement along the symphysis [14]. Importantly, the transfacial pin facilitates oral excursion and early movement at the TMJ after removal of the MMF wires without compromise of rigid fixation. Oral excursion exercises and mandibular loading can occur with the Steinman pin in place. The pin can be removed after 8 weeks, when bony union has completed. To the authors’ knowledge, no other transfacial pin fixation has been described in the pediatric patient population for facial trauma, aside from the senior author’s prior case report. In the adult population, there is documentation of Steinman pins being utilized for mandibular reconstruction with no reported complications [34].

Recently, Yesantharao et al. conducted a nearly 30-year retrospective review of pediatric condylar-symphyseal mandible fractures [7]. In this cohort of 21 patients, the overall complication rates were 62.5 percent in open reduction and internal fixation patients, 14.3 percent in closed treatment patients, and 16.7 percent in patients treated with soft diet only [7]. Furthermore, Yesantharao et al. proposed a treatment algorithm based on displacement and dentition stage to maximize outcomes in patients with these fracture patterns [7]. Interestingly, in the deciduous patient category of the algorithm, as applicable to our 3-year-old patient presented here, this displaced combined symphyseal-condylar fracture would be managed with closed reduction only, which may not fully address the bony displacement commonly seen in this fracture pattern. This may account for the 20 percent post-treatment complication rate seen in the patients treated in this manner [7].

The technique reported therein is new and long-term follow up and additional surgical experience will be required to elucidate its benefits and limitations. Potential challenges with the described technique include: facial nerve damage, puncture of the endotracheal tube, injury to the branches of the external carotid artery, infection, facial scarring, injury from the transfacial pin itself (namely, the patient scratching oneself on the prominent aspect of the pin *via* accidental removal of the pin dressing), and inappropriate reduction after fixation. It is notable that this staged surgery required three visits to the operating room. Although the latter two were brief (for hardware removal) they account for additional anesthetic exposures nonetheless and this should be considered when pursuing this procedure. Intraoperative imaging guidance and radiographic image capture can facilitate placement of the pin and confirmation of bony reduction in future cases. This technique can provide stable reduction of a bilateral condylar tripartite fracture with lateral dislocation of the TMJ, a challenging and uncommon fracture pattern affecting children.

## Conclusion

Transfacial Steinman pin with circummandibular wiring can successfully treat pediatric bilateral condylar and symphyseal fractures with lateral dislocation of the TMJ. This transfacial pinning technique can be a safe, viable option for management of these tripartite pediatric mandibular fractures.
